# EDL-360: A Potential Novel Antiglioma Agent

**DOI:** 10.4172/1948-5956.1000295

**Published:** 2014-09-25

**Authors:** Amira Hosni-Ahmed, Michelle Sims, Terreia S Jones, Renukadevi Patil, Shivaputra Patil, Hossam Abdelsamed, Charles R. Yates, Duane D Miller, Lawrence M Pfeffer

**Affiliations:** 1 Departments of Pathology and Laboratory Medicine, University of Tennessee Health Science Center, USA; 2 The Center for Cancer Research, University of Tennessee Health Science Center, USA; 3 Department of Pharmaceutical Sciences, University of Tennessee Health Science Center, Memphis, USA; 4 Scientific Affairs, Amgen, Thousand oaks, CA, USA; 5 Department of Immunology, St. Jude Children’s Research Hospital, Memphis, TN, USA; 6 Department of Chemistry, College of Science, Fayoum University, Fayoum, Egypt

**Keywords:** Ovarian cancer, PARP inhibitors, Angiogenesis

## Abstract

Glioma is a brain tumor that arises from glial cells or glial progenitor cells, and represents 80% of malignant brain tumor incidence in the United States. Glioblastoma multiforme (GBM) is the most aggressive primary brain tumor malignancy with fewer than 8% of patients with GBM surviving for more than 3 years. Over the past 10 years, despite improvement in diagnosis and therapies for cancer, the survival rate for high-grade glioma patients remains dismal. The main focus of our research is to identify potent novel antiglioma small molecules. We previously showed that EDL-360, a tetrahydroisoquinoline (THIQ) analog, as being highly cytotoxic to human glioma cell cultures. Here we show that EDL-360 significantly induced apoptosis in human glioma cell lines (U87 and LN18). However, in normal astrocytic cells, EDL-360 induced a modest G0/G1 cell cycle arrest but did not induce apoptosis. In an attempt to enhance EDL-360 induced cell death, we tested simultaneous treatment with EDL-360 and embelin (an inhibitor of the anti-apoptotic protein, XIAP). We found that, glioma cells had significant lower viability when EDL-360 and embelin were used in combination when compared to EDL-360 alone. We also used combination treatment of EDL-360 with decylubiquinone (dUb), a caspase-9 inhibitor, and found that the combination treatment induced a significant cell death when compared to treatment with EDL-360 alone. This is the first report that suggests that dUb has anticancer activity, and perhaps acts as a XIAP inhibitor. Finally, our *in vivo* data showed that EDL-360 treatment induced a partial regression in glioma tumorigenesis and induced cell death in the treated tumors as shown by H&E staining. Taken together these data suggests that EDL-360 has a potential therapeutic application for treating glioma, especially when combined with XIAP inhibitors.

## Introduction

According to the Central Brain Tumor Registry of the United States (CBTRUS) report, gliomas represent 80% of the malignant brain tumor incidence in the United States [1]. The worldwide incidence of malignant brain tumors is approximately 3 in 100,000 per year, with a higher rate in the developed countries compared to the developing countries [2,3]. Grade IV glioma, glioblastoma multiforme (GBM), is the most aggressive subtype of brain tumor. It has a dismal prognosis with a median survival of ~15 months, and is characterized by diffuse infiltration into normal brain parenchyma, which makes tumor resection very challenging. Consequently, these tumors can repopulate after surgery, causing cancer relapse [4]. The standard treatment involves surgical resection followed by cranial irradiation, which can alleviate the neurological symptoms and seizures in glioma patients. Despite aggressive therapeutic approaches such as radiation and chemotherapy, there has not been a significant improvement in either the prognosis or the survival of patients for several decades. Therefore, there is an urgent need to find new therapeutic approaches to improve patient survival and outcome. We previously showed that tetrahydroisoquinoline (THIQ) derivatives possessed potent antiglioma activity [5]. Structure activity relationship studies showed that EDL-360 is the most potent THIQ derivative against glioma cell cultures. The IC_50_ values ranged from approximately 5–10 μM in multiple cancer cell lines [5].

Inhibitors of apoptosis proteins (IAPs) are key regulators of apoptotic cell death as well as the necrosis pathway. In addition, IAPs regulate cell cycle progression and inflammatory response [6]. X-chromosome-linked IAP (XIAP) was the first identified IAP protein and is directly involved in caspase regulation. In particular, XIAP binds with high affinity to caspases 3, 7 and 9 leading to their inactivation [7]. XIAP overexpression leads to caspase inhibition and consequently can confer resistance to chemotherapy/radiotherapy in cancer. Not surprisingly, overexpression of XIAP has been observed in many cancer types including glioma, leukemia, melanoma and carcinoma [8–11]. Giving this crucial role in regulating cell death and cancer, XIAP proteins have been very attractive targets for cancer treatment. Indeed, *in vitro* targeting of XIAP protein sensitized glioma cells to radiotherapy and induced apoptotic cell death [12]. Here we show that EDL-360 induces apoptotic cell death in glioma cultures. In attempt to enhance the cytotoxic action of EDL-360, we investigated the effect of simultaneous activation of the caspase pathway and EDL-360 treatment on cell viability. We used human glioma cultures as well as normal astrocytic cultures to address this question. In addition, we used a mouse glioma mouse model to examine the anticancer activity EDL-360 *in vivo*.

## Materials and Methods

### Drug preparation

EDL-360 was prepared as previously described [5]. For *in vitro* screening, a freshly prepared 50 μM stock solution was prepared by dissolving EDL-360 in DMSO. For treatment of tumor-bearing mice, EDL-360 was dissolved in 0.9% saline. Embelin and decylubiquinone (dUb) were dissolved in DMSO to prepare a 100 μM stock solution.

### Glioma cell lines and primary astrocyte cultures

The human glioma cell lines, U87 and LN18, were obtained from the American Type Culture Collection Manassas, VA, USA). U87 cells were grown in Minimum Essential Medium Eagle (Cellgro, Hemdon, VA), supplemented with 10% fetal bovine serum, 2 mM L-glutamine, 0.1 mM nonessential amino acids, 1 mM sodium pyruvate, 100 IU/ml penicillin, and 100 μg/ml streptomycin. LN18 cells were grown in Dulbecco’s Modified Eagle’s Medium (Cellgro, Hemdon, VA), supplemented with 10% fetal bovine serum, 2 mM L-glutamine, 100 IU/ml penicillin, and 100 μg/ml streptomycin. All cell cultures were maintained at 37°C in a 5% CO_2_ humidified atmosphere.

Astrocyte cultures were established from 2–5 day old mice. A dissecting microscope was used to remove the meninges and hippocampus, and the cortices were mechanically dissociated. Subsequently, cultures were established and maintained in a 50:50 mix of Dulbecco’s Modified Eagle’s Medium/Ham’s F-12 (Cellgro, Hemdon, VA) supplemented with 10% fetal bovine serum, 2 mM L-glutamine, 100 IU/ml penicillin, 100 μg/ml streptomycin and 20 ng/ml epidermal growth factor (Millipore Co., Bedford, MA) and grown in Primaria flasks (BD Bioscience, San Jose, CA). After three days of incubation, cultures were supplemented with 20 ng/ml epidermal growth factor, and the media was changed after five days. At passage 2, three individual primary cultures were pooled and seeded for experiments. Only cultures with greater than 80% astrocytes (as determined by GFAP immunohistochemistry) were used in experiments.

### Cell viability assay

Cell viability was measured using 3-(4,5-dimethylthiazol-2-yl)-2,5-diphenyl tetrazolium bromide (MTT) (Sigma-Aldrich, St. Louis, MO). In brief, 1000 cells were seeded per well in 96-well plates. Plates were pre-coated with poly-L-ornithine (Sigma-Aldrich, St. Louis, MO) and laminin (Invitrogen, Carlsbad, CA) for primary astrocytes to facilitate cell attachment. Cells were allowed to attach overnight and small molecules or vehicle control was added to the appropriate wells. After five days, MTT was added and incubated for 3 hr. The media was carefully aspirated and the purple formazan crystals were dissolved in 100 μl DMSO (Fisher Scientific Co., Fair Lawn, NJ). Absorbance was measured at 570/690 nm using a FLx800 fluorescence microplate reader (BioTek Instruments, Inc., Burlington, VT). Experiments were conducted to determine the half-maximal inhibitory concentration (IC_50_) for each compound.

### Cell cycle analysis

The effect of EDL-360 on cell cycle phase distribution was determined by flow cytometry. Cells were seeded at a density of 9,000 cells per 10 cm plate, allowed to attach to the plates overnight, and then treated with 20 μM EDL-360 for 3 days. After trypsinization and washing in ice cold PBS, the cells were fixed in ice cold 70% ethanol for at least 1 hr. Subsequently, the cells washed with PBS and stained with 0.05 mg/ml propidium iodide (PI) in presence of 0.1 mg/ml RNAses and then analyzed using BD Accuri C6 flow cytometer (BD Bioscience, San Jose, CA). The different phases of cell cycle (G0/G1, S and G2/M) as well as the percentage of apoptotic cells of sub-G1 population were quantified using FlowJo software - v7.6.5 (Verity Software House, Tosham, ME).

### Lentiviral transduction

Luciferase cDNA was amplified by PCR, purified through agarose gel electrophoresis, and subcloned into BamHI and EcoRI sites of pLenti-pgk-puro vector (obtained from Viral Vector Core, UTHSC). The pLenti-Luc-mKate-pgk-puro and pLenti-pgk-puro lentiviral (empty vector for mock transfection) vectors were packaged in 293T cells [13]. The lentiviral vectors were used to transduce the U87 glioma cell line in the presence of 6 μg/mL polybrene (Sigma-Aldrich, St. Louis, MO). Pools of stably transfected cells (U87^Luc^ and U87^Mock^) were then selected by growing the cells in 1 μg/ml puromycin. Transduction efficiency was determined by using Nikon Eclipse TE300 fluorescent microscope.

### Luciferase activity quantification

Luciferase activity was measured in U87 transduced cells using dualluciferase reporter assay (Promega, Madison, WI). U87^Luc^ and U87^mock^ were seeded in 6-well plates and allowed to reach 70% confluency. After removing cell medium and washing once with PBS, the cells were lysed in 500 μL 1X passive lysis buffer (PLB) for 15 min at room temperature with gentle shaking. Subsequently, 10 μL of PLB lysate was mixed with 50 μL LARII (luciferase substrate) and firefly luciferase activity was determined using GloMax-Multi Jr Single-Tube Multimode Reader (Promega, Madison, WI). Mean luciferase luminescence was 2.1×10^7^ relative light units (RLU) per 10^6^ U87^Luc^ cells (± SD 6.4×10^5^).

### U87 glioma xenograft

Animal experiments were performed in accordance with a study protocol approved by the Institutional Animal Care and Use Committee of the University of Tennessee Health Science Center. A glioma xenograft mouse model was generated by injecting U87^Luc^ cells subcutaneously in 4–6 week old NSG (NOD.Cg-Prkdc**^scid^** Il2rg**^tm1Wjl^**/SzJ) mice (Jackson Laboratory, Bar Harbor, ME). The mice were anesthetized using isoflurane and subcutaneously injected with 100 μL containing 10^7^ U87^Luc^ cells in the right flank of the mice. Once the tumors were palpable, the mice were divided randomly into groups of 5 mice. Subsequently, 5 μM of EDL-360 was administered intratumorally. Tumor size and body weight were obtained twice/week. In addition, non-invasive bioluminescent imaging was acquired biweekly using the IVIS-200 Imaging System (Xenogen Corporation, Berkeley, CA). The mice were euthanized by carbon dioxide when the tumors reached 10% of total body weight.

### Histology

Tumor tissue was harvested and fixed in 10% neutral buffered formalin solution (Sigma-Aldrich, St. Louis, MO) for 24 to 72 hr. After fixation, tissue samples were paraffin embedded for sectioning (4 μM thick) and stained with Hematoxylin and Eosin (H&E).

### Statistical analysis

Mann-Whitney *U* test was used to compare between two groups. The results were considered significant when p<0.05. All statistical analyses were performed using Statistica 8 (StatSoft Inc., Tusla, OK).

## Results

### EDL-360 treatment induced apoptosis in glioma cultures but not in normal astrocytes

**Figure 1:**
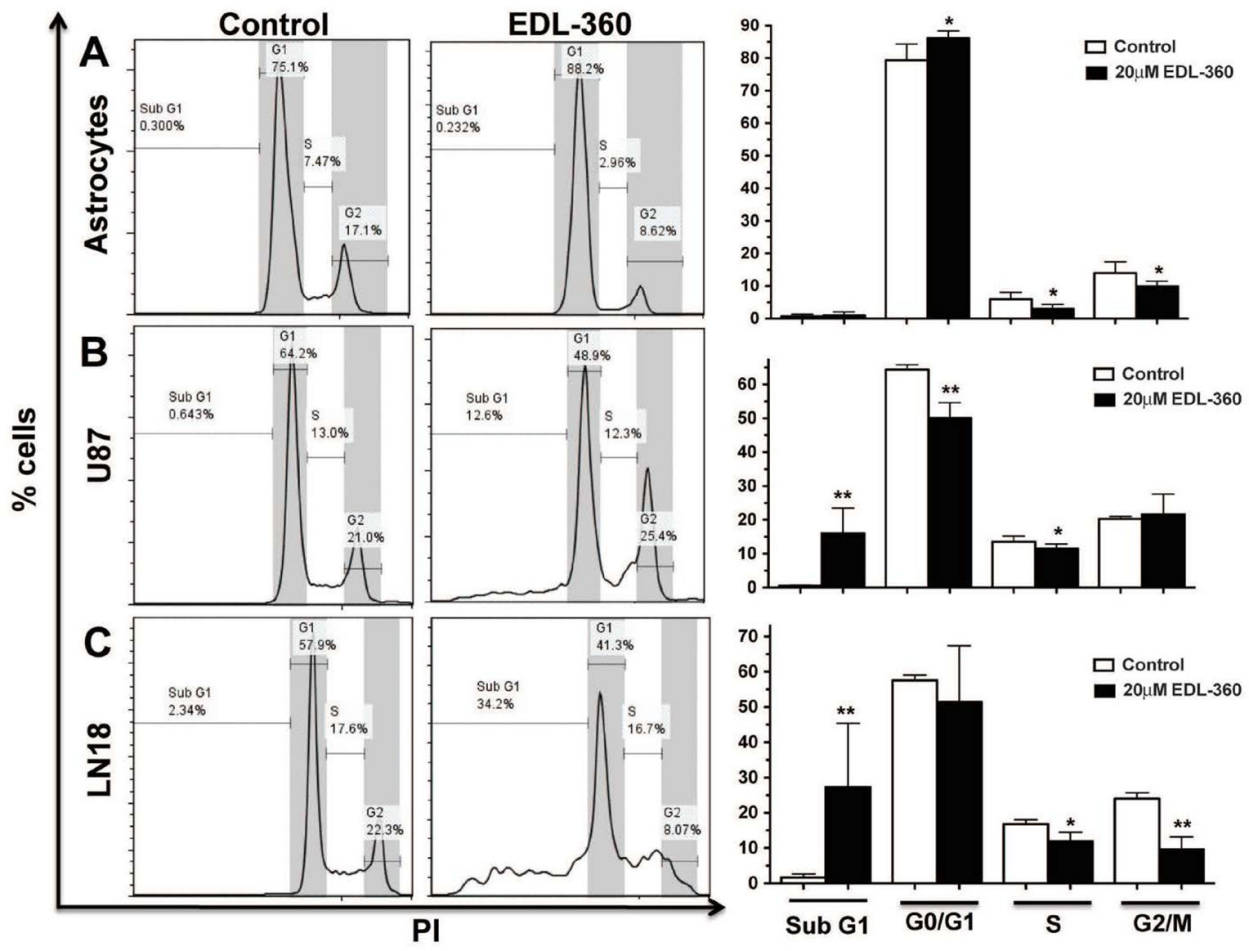
Cell cycle analysis of EDL-360 treated glioma and normal astrocytic cells Normal astrocytic cell cultures (**A**), U87 (**B**) and LN18 (**C**) glioma cell cultures were treated for 3 days with 20 μM EDL-360. The cells were fixed and then stained with PI and analyzed on an Accuri C6 flow cytometer. Cells undergo apoptosis were identified by SubG1 quantified. Bars represent the means; whiskers represent the standard deviation (±); * p<0.05 and ** P<0.005].

The effect of EDL-360 on cell cycle arrest and induction cell death was determined by flow cytometry. U87 and LN18 glioma cultures as well as normal mouse astrocytes were treated with 20 μM of EDL-360 for 3 days and the distribution of cell cycle phase was quantified. Although EDL-360 did not induce significant cell death in normal astrocytes, it caused a modest cell cycle arrest at G0/G1 phase of the cell cycle (Figure 1A). In addition, there was a significant decrease in S and G2/M phases (p=0.037) compared to untreated astrocytic cultures. In contrast, glioma cell cultures were much more sensitive to EDL-360 treatment compared to normal astrocytes. Furthermore, there was a significant increase (p=0.0039) in the fraction of apoptotic (sub-G1 population) in EDL-360 treated U87 and LN18 glioma cultures compared to the control cultures (Figures 1B and 1C). Taken together, these results demonstrated that EDL-360 induced apoptotic cell death in human glioma cell lines. Consequently, we next examined whether activating the caspase pathway and/or blocking the activity of caspase inhibitors, could augment EDL-360 induced cell death.

### Simultaneous treatment with embelin and EDL-360 induced cell death in glioma cultures

**Figure 2:**
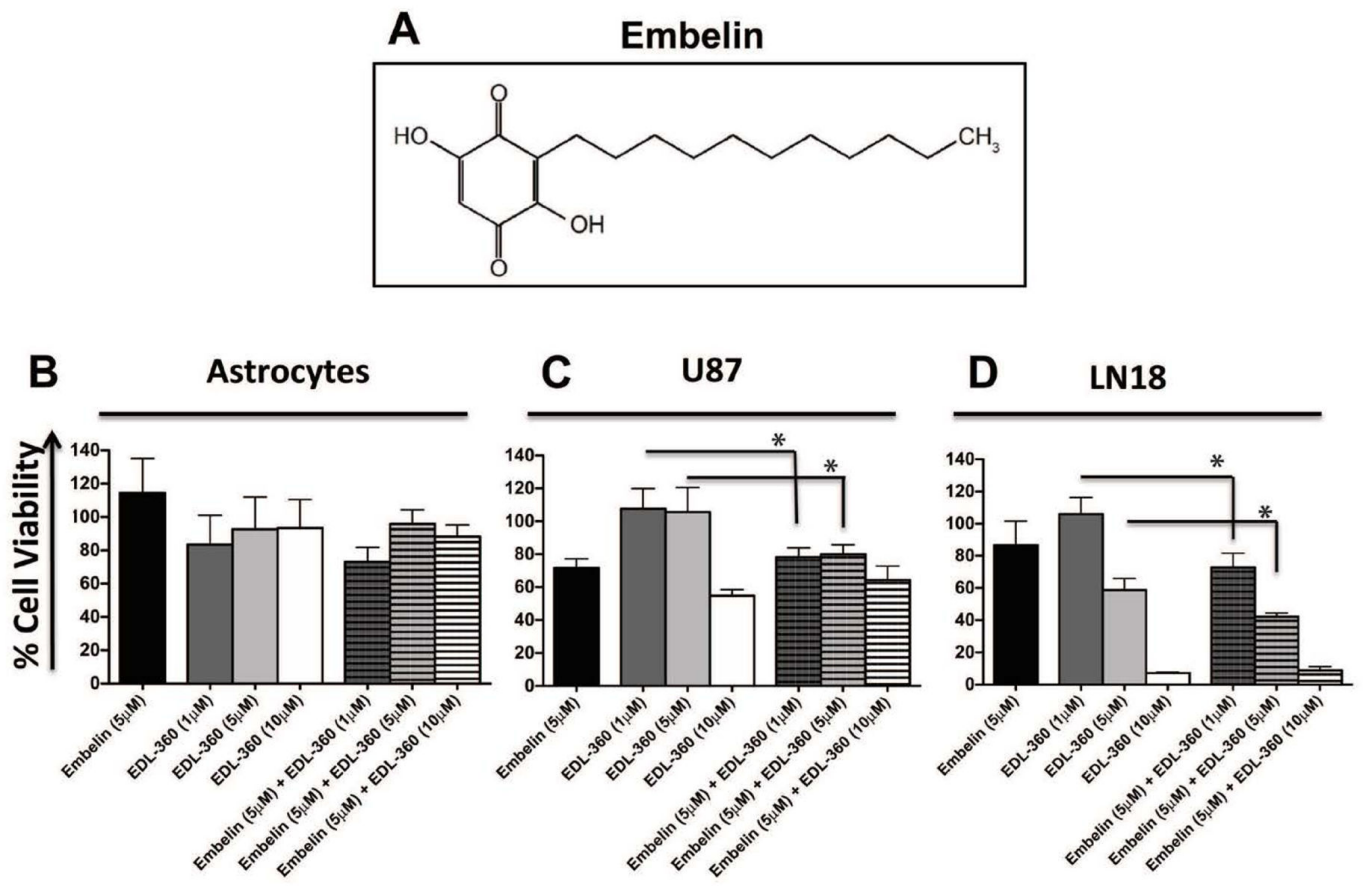
Simultaneous treatment with Embelin and EDL-360 induced cell death in glioma cultures Glioma and normal astrocytic cell cultures were plated in 96-well plates and treated with either EDL-360 (1, 5, 10 μM) or 5 μM embelin as a single treatment or in combination. The cultures were incubated with the drug for 5 days and cell viabilities were measured by MTT assay. (**A)**, Chemical structure of embelin. Viable astrocytes (**B**), U87 cells (**C**) and LN18 cells (**D**) were calculated as a percentage of vehicle treated cells. Bars represent the means; whiskers represent the standard deviation (± SD); * p<0.05.

Embelin has recently been identified as a XIAP inhibitor and induces caspase-dependent apoptosis in leukemic cells [14]. Therefore, we treated glioma and normal astrocyte cell cultures with EDL-360 as a single agent (1, 5, 10 μM) or in a combination with 5 μM embelin, and determined cell viability using MTT assay. Glioma cultures treated with embelin had a slightly lower viability (80% viability) compared to normal astrocytes (~100% viability). In agreement with the previously published data [5], LN18 was the most sensitive glioma culture to EDL-360 compared to normal astrocytes. The combination of 1, 5 or 10 μM EDL-360 and 5 μM embelin had no significant effect on normal astrocyte cell viability (p=0.09, p=0.73, p=0.83, respectively) (Figure 2B). However, treatment with either 1 or 5 μM of EDL-360 and embelin had a significant effect on the viability of U87 (p=0.014 and p=0.011, respectively) (Figure 2C and 2D). The higher sensitivity of LN18 cells was reflected in the lower cell viability when the cultures co-treated with 1 or 5 μM of EDL-360 and embelin (p=0.011 and p=0.0066, respectively) (Figure 2C and 2D). Surprisingly, in both U87 and LN18 glioma cultures, there was no significant difference in cell viability between 10 μM EDL-360 alone and in combination with embelin (p=0.09 and p=0.39, respectively) compared to cultures treated with EDL-360 alone.

### Combination treatment with dUb and EDL-360 induce synergetic cell death in glioma cultures

**Figure 3:**
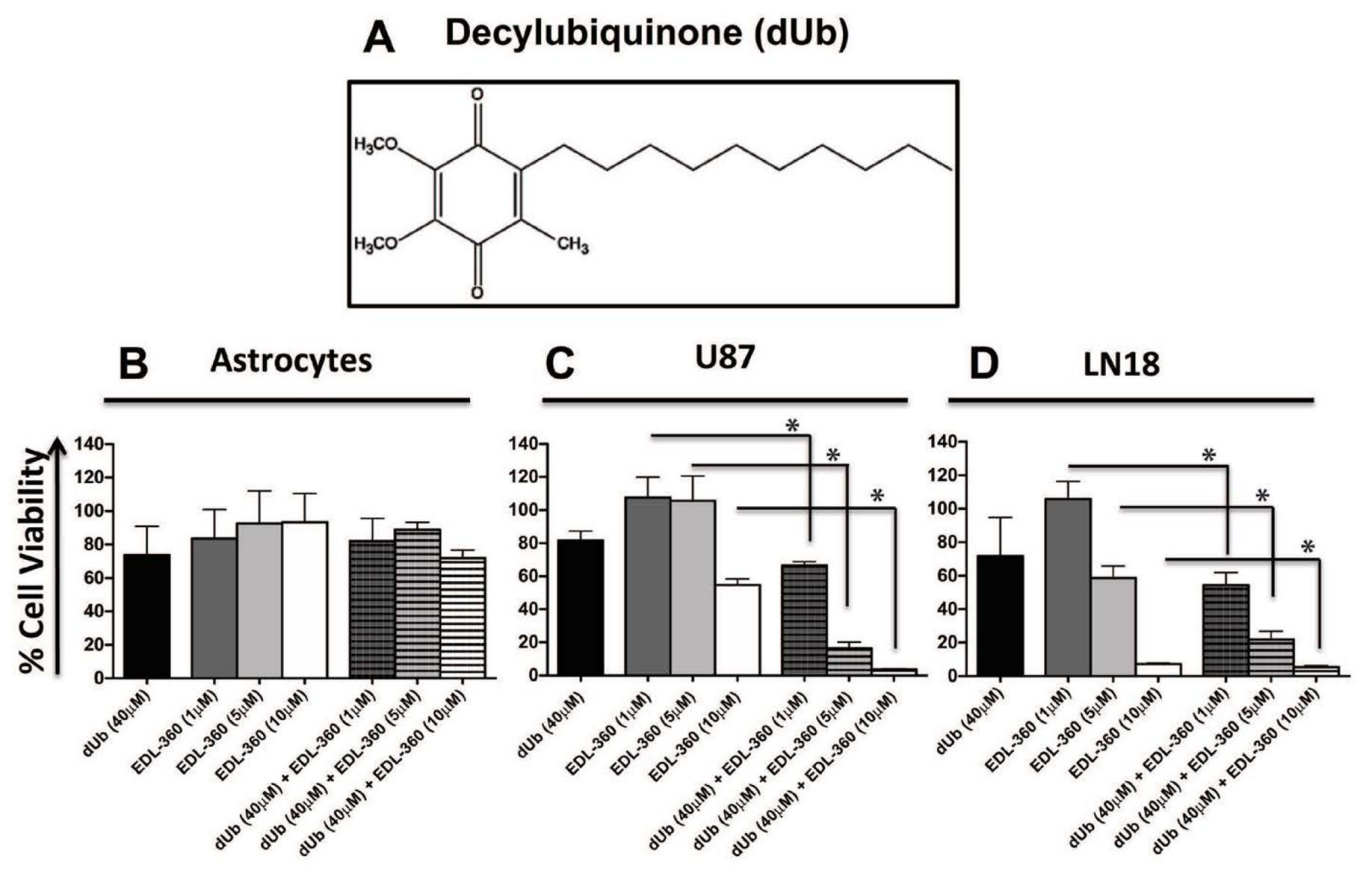
Combination treatment with dUb and EDL-360 induce synergetic cell death in glioma cultures Glioma and normal astrocytic cultures were plated into 96-well plates and treated with either EDL-360 (1, 5, 10 μM) or 40 μM dUb as a single treatment, or in combination. The cultures were incubated with the drug(s) for 5 days and cell viabilities were measured by MTT assay. (**A)**, Chemical structure of dUb. Viable astrocytes (**B**), U87 (**C**) and LN18 (**D**) were calculated as a percentage of vehicle treated cells. Bars represent the means; whiskers represent the standard deviation (±SD); * p<0.05.

Since EDL-360 induced the apoptosis of glioma cells and this effect was augmented when EDL-360 treatment was combined with a XIAP inhibitor (embelin), we investigated the effect of the combination treatment with an inhibitor of apoptosis (decylubiquinone, dUb). dUb was previously shown to inhibit caspase 9 activation and PARP cleavage [15]. Glioma and normal astrocyte cultures were treated with EDL-360 alone (1, 5, 10 μM) or in combination with 40 μM dUb. dUb treatment in combination with EDL-360 did not have a significant effect on astrocyte cell viability at 1, 5 or 10 μM (p=1, p=1, p=0.54) (Figure 3A). Surprisingly, combination treatment with both compounds induced enhanced cell death in glioma cultures. U87 and LN18 cells that were treated with 1 μM EDL-360 in combination with dUb had >35% and 45% cell death compared to treated cells with EDL-360 alone (p=0.007 and p=0.006, respectively) (Figure 3C and 3D). While combined treatment with 5 μM EDL-360 and dUb caused 84% and 63% cell death in U87 and LN18 (p=0.007 and p=0.007, respectively) when compared to EDL-360 treatment alone. Finally, combination treatment with 10 μM EDL-360 and dUb induced 93% and 27% cell death in U87 and LN18 cells, respectively, compared to EDL-360 treatment alone (p=0.011 and p=0.03, respectively).

### EDL-360 reduces tumor growth in human glioma xenograft mouse model

**Figure 4:**
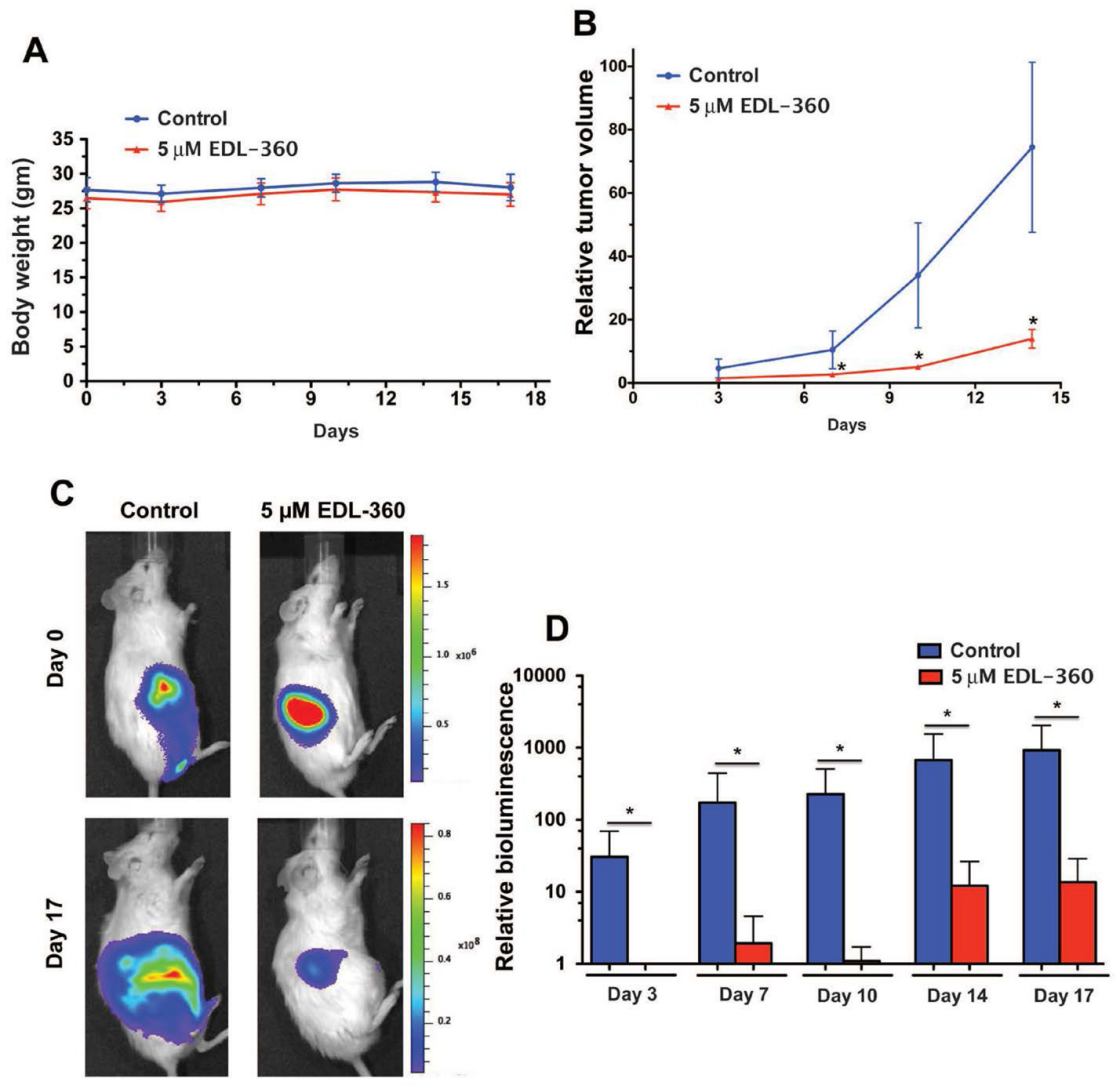
EDL-360 reduces tumor growth in human glioma xenograft mouse model U87^Luc^ cells were injected into the right flank of the mice. Once the tumor became palpable in the mice, treatment was initiated. **A**, Weight of the mice was obtained twice a week. There was no sign of toxicity or weight lose in EDL-360 treated group compared to the control mice. **B**, Tumor dimensions were obtained using digital caliper to determine tumor volume. **C**, Bioluminescence signal from control and treated mice showed an obvious decrease in tumor viable cells after 17 days of treatment. **D**, Quantification of bioluminescence signal showed a continuous tumor growth in the control mice. Bars represent the means; whiskers represent the standard deviation (±); * p<0.05.

To assess the antiglioma activity of EDL-360 *in vivo*, mice bearing U87 glioma xenografts were treated with a daily single dose of the compound, and tumor growth examined by caliper and Xenogen animal imaging. For bioluminescence imaging, the U87 glioma cell line was transduced with the pLenti-Luc-mKate-pgk-puro viral vector, and the cells were grown in puromycin to select stably transfected cells. To generate the glioma mouse model, U87^Luc^ cells were injected subcutaneously into the right flank of 4–6 week old BALBc (NSG) mice. Once the tumor became palpable, the mice were randomly assigned into groups of 5 mice. The treated group received 5 μM of EDL-360 daily, whereas the control group did not receive any treatment. Mice were monitored daily for any signs or symptoms of drug related toxicity. In addition, mice were weighed twice a week. There was no sign of toxicity or weight loss in the EDL-360 treated group compared to the control mice (Figure 4A).

Tumor dimensions (width, length and height) were obtained using a digital caliper to determine tumor volume (mm^3^). Tumor volume of each mouse was normalized to the initial volume before starting EDL-360 treatment (day 0). A slight attenuation of tumor growth was observed as early as three days post-treatment (p=0.077) (Figure 4B). The tumors in the EDL-360 treated mice were significantly smaller compared to control mice after 7, 10 and 14 days of treatment (p=0.03, 0.02, and 0.02, respectively). The difference in tumor size between EDL-360 treated tumors and the controls increased with time of treatment. For instance, at three days after treatment the EDL-360 treated tumors were 1/3 the size of the control group (Mean of 1.5 vs. 5 normalized tumor volume). After 14 days of treatment, the treated tumors were 1/6 the size of control group (Mean of 12.5 vs. 74 normalized tumor volume). In addition, non-invasive live imaging was employed to evaluate tumor growth in EDL-360 and control mice. Bioluminescence signal showed a significant decrease in viable tumor cells at 17 days of treatment compared to the controls (Figure 4C). Quantification of the bioluminescent signal showed a continuous tumor growth in the control mice (Figure 4D). In addition, EDL-360 treated tumors had a significantly lower growth rate compared to the controls (P<0.03).

**Figure 5:**
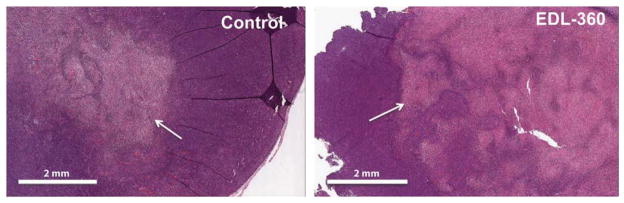
Histopathological analysis of EDL-360 treated gliomas Paraffin embedded tumor sections from control and EDL-360 treated mice were stained with H&E for histopathological analysis. White arrows indicate necrotic regions (Pink areas).

Histopathological analysis of H&E stained tumor samples from control and EDL-360 treated group showed that EDL-360 induced necrosis within the tumor (Figure 5, white arrows). In particular the mean area of necrosis in untreated animals is 28.6 μm^2^ (± 25.7) whereas in EDL-360 treated animals is 45.9 μm^2^ (± 21.5).

## Discussion

Despite aggressive therapeutic approaches to treat glioma, there is no significant improvement in either the prognosis or the survival of patients. Therefore, there is an urgent need to find new therapeutic approaches to improve patient survival and outcome. We previously identified EDL-360 as a potent antiglioma compound with low toxicity to normal brain stromal cells [5]. Interestingly, it appears that EDL-360 exhibited a selective toxicity towards LN18 (TMZ resistant cell line) and A172 cells when compared to T98 and U87 (TMZ sensitive cell line) cells raising the potential use of this analog in the clinical setting for TMZ resistant tumors [5].

The current study focused on assessing the potential anticancer activity of THIQ analog, EDL-360. Several groups reported cytotoxic activity of different THIQ derivatives against numerous cancer types including retinoblastoma, squamous call carcinoma, lung cancer cells and breast cancer [16–20]. The antiproliferative properties of THIQ may in part reflect its ability to inhibit microtubule polymerization [21–23]. Our data suggests that EDL-360 induced cell death via an apoptotic pathway in glioma cells as measured by the accumulation of sub-G1 population (Figure 1). It is plausible that apoptotic cell death is a consequence of inhibiting tubulin formation. Interestingly, EDL-360 apparently exhibits two different mechanisms of cytotoxicity. For example, in normal cells EDL-360 induced a modest G1 cell arrest without detectable levels of cell death. However, in GBM cell lines EDL-360 induced apoptotic cell death, which indicates the high specificity of the novel compound towards cancer cells.

Since EDL-360 induced apoptosis in glioma cultures, we considered whether simultaneous treatment with EDL-360 and embelin (a XIAP inhibitor) would increase the potency of EDL-360 compound. XIAP up-regulation could contribute to tumorigenicity and XIAP has been observed to be over-expressed in numerous types of cancers [12,24,25]. Indeed, combination treatment of embelin with EDL-360 resulted in a significant decrease of cell viability compared to cells treated with EDL-360 alone. In attempt to further elucidate the mechanism of action of EDL-360, we investigated the effect of combination treatment with EDL-360 and dUb on the viability of glioma and normal astrocytes. dUb is a potent inhibitor of mitochondrial permeability transition (MPT), and hence blocks the release of cytochrome C from the mitochondria and inhibits apoptosis [26]. In addition, dUb was found to inhibit caspase 9 activation and PARP cleavage [15]. Therefore, we anticipated that dUb would abrogate the cytotoxic action of EDL-360. However, combination treatment of EDL-360 and dUb also had a synergistic cytotoxic effect. One possible explanation is that dUb exhibits a fairly similar chemical structure to emblin, and might act as a XIAP inhibitor in brain tumor cells. To our knowledge this is the first publication suggesting that dUb might be an inhibitor of XIAP, and the combination of dUb with THIQ potentiates its anticancer activity. In addition, our data show that combination treatment with dUb and EDL-360 resulted in a similar toxic effect in both TMZ sensitive (U87) and resistant cancer cell type (LN18), which may have important clinical implications, since TMZ is the mainstay of treatment for glioma. Previously published data suggested that EDL-155, THIQ analog, induced autophagy in retinoblastoma cancer cells [18]. However, there are increasing number of articles suggesting that caspases orchestrate apoptotic as well as autophagic cell death pathways [27,28]. Indeed, a recent study reported that caspase 9 plays a pivotal role in the induction of autophagy via autophagosome formation [27]. It is plausible that caspase 9 is the molecular target of EDL-360 compound, however future experiments are required to identify the molecular target(s) of EDL-360.

The *in vivo* anticancer activity of EDL-360 was further tested in glioma-bearing mice generated by injecting human glioma U87^Luc^ cells subcutaneously in immunocompromised mice. Although intracranial injection of U87 cells into mice produces glioma characterized by leaky Blood Brain Barrier (BBB) similar to human glioma, the tumor does not have infiltrative nature [29]. There was no sign of EDL-360 induced toxicity as indicated by the absence of weight loss in the EDL-360 treated group compared to the control mice. The tumors of EDL-360 treated mice were significantly smaller compared to control mice. In addition EDL-360 treated tumors showed large areas of cell death and necrosis. It would be interesting to investigate whether EDL-360 mediates inflammatory response by regulating the expression of proinflammatory mediators such as iNOS, IL-6 and TNF-α. However, it is still unclear whether EDL-360 penetrates the BBB and would exert a similar anticancer activity when the tumors are grown intracranially. Previous studies showed that EDL-155 (THIQ analog) could penetrate the BBB, with 1.4% brain to plasma exposure ratio [30]. Slight variation in the chemical structure could lead to major changes in the biological responses. Probing the structure activity relationship of EDL-360 may improve its anticancer activity. In addition, further investigations are required to test the anticancer activity of this new small molecule in an intracranial xenograft model, which would allow for a more reliable assessment of the antiglioma activity in the CNS.

In conclusion, EDL-360 is a potential novel antiglioma compound that showed highly toxic *in vitro* as well as decreasing tumor size in an *in vivo*. Further studies are necessary to optimize and test the effect of EDL-360 in combination with dUb on tumor growth *in vivo* perhaps in a more clinically relevant glioma mouse model.
